# Are You Sure: Preference and Ambivalence in Delay Discounting

**DOI:** 10.3389/fnbeh.2021.782991

**Published:** 2022-01-24

**Authors:** Sergej Grunevski, Aaron P. Smith, Richard Yi

**Affiliations:** Cofrin Logan Center for Addiction Research and Treatment, University of Kansas, Lawrence, KS, United States

**Keywords:** ambivalence, choice, preference, delay discounting, Monetary Choice Questionnaire

## Abstract

Delay discounting (DD) research has become ubiquitous due to its robust associations with clinical outcomes. Typical DD tasks involve multiple trials in which participants indicate preference between smaller, sooner and larger, later rewards. Scoring of these binary choice tasks has not considered trial-level ambivalence as a possible decision-making construct. The present study explored the extent to which trial-level ambivalence varied within-individual using an established assessment of DD (the Monetary Choice Questionnaire). Results indicate that degree of ambivalence peaks around the trials associated with the DD rate. Moreover, ambivalence is associated with a diminished impact of reward delay differences on choice, where greater delay differences decrease the odds of choosing the larger, later rewards. Taken together, we believe ambivalence to be a relevant construct for research on intertemporal decision making, and it may be particularly useful in the study of manipulations on individual rates of DD.

## Introduction

Delay discounting (DD) refers to the reduction in the subjective value of an outcome when its delivery is delayed (Odum, 2011), and a substantial body of literature has linked rates of DD to behaviors where immediate rewards have delayed consequences. For instance, higher rates of DD, indicating steeper reductions in subjective value across increasing delays to reward receipt, are associated with substance misuse (Yi et al., 2010; MacKillop et al., 2011) and poor treatment outcomes (MacKillop and Kahler, 2009; Sheffer et al., 2012; Stanger et al., 2013), risky sexual behavior (Chesson et al., 2006), overeating (Kekic et al., 2020), and other behaviors associated with similar intertemporal trade-offs.

Common procedures for assessing DD are multi-trial binary choice tasks where the individual indicates preference between smaller, sooner rewards (SSs; usually money) and larger, later rewards (LLs; also money). Though the manner of determining the index of DD (i.e., scoring) varies by task, most variations of these binary choice tasks summarize the pattern of choices across all trials to determine a *rate* of DD, e.g., the *k* value per Mazur’s (1987) hyperbolic discounting equation. For instance, the Monetary Choice Questionnaire (MCQ; Kirby et al., 1999) is a 27-trial binary choice task where participants are presented fixed pairs of immediate/delayed outcomes and asked to indicate the preferred outcome in each trial (e.g., “Would you prefer $15 today, or $35 in 13 days?”). A DD rate obtained from the MCQ reflects the approximate point of switching from preferring the SSs to LLs when trials are placed in rank order of associated DD rate (i.e., the discount rate at which the SS and LL are of equal value). However, an implicit assumption not explicitly stated in scoring these tasks is that an individual has a constant degree of certainty (conversely, ambivalence) in their preference across trials. That is, it is assumed that a participant who prefers *$34 today* rather than $*35 in 186 days* is equally certain in their preference for *$15 today* rather than *$35 in 13 days*. We hypothesize that this assumption is likely incorrect. That is, as the immediate/delayed outcomes approach values of subjective equivalence at an individual level, the decisions become more difficult and increase the degree of ambivalence about the participant’s preference.

Previous efforts have sought to examine this possibility using behavioral proxies for the ambivalence construct. As it is intuitive for a greater degree of deliberation to occur as the subjective values of two options approach equivalence, one might expect that the deliberation period increases as the outcomes become subjectively equivalent at the individual level. Multiple studies examining choice reaction times (RTs) in DD tasks as the indirect measure of ambivalence have found that RTs tend to be longest on trials around the point of subjective equivalence (Robles and Vargas, 2007, 2008; Rodriguez et al., 2014). Moreover, a study examining mouse cursor trajectories in similar tasks discovered that trials around the point of subjective equivalence (termed *indifference point* in other DD assessments; Mazur, 1987) were associated with significantly greater mouse curvatures and, by implication, deliberation (Dshemuchadse et al., 2013). As these studies examined behavioral proxies for ambivalence, the current study sought to explore decision making as it relates to choice difficulty *via* degree of participant-reported ambivalence on each trial of a binary choice DD task. Defining *ambivalence* as the state of indecision toward an attitude (in this case, preference), we proposed to evaluate within-individual variability in degree of self-reported ambivalence across trials in the MCQ. We used previous research on ambivalence as a starting point (Priester and Petty, 1996, 2001) to develop four different assessment strategies. Within the MCQ, and individual’s *k* value represents the approximate point where they switch from preferring SSs to LLs. Stated differently, along the continuum of MCQ trials, the *k* value ostensibly denotes the point of equivalence between SSs and LLs of the surrounding trials; as such, degree of ambivalence should steadily increase toward and peak around this “switch point.” Our overall hypothesis was that ambivalence would vary across MCQ trials and, specifically, that the (H1) within-individual variability in ambivalence would track switches in preference (i.e., ambivalence peaks around switch point).

In addition to discount rates, another means of analyzing discounting data is *via* how “sensitive” a participant is to the relative differences in reward delays and magnitudes of both choice options (Wileyto et al., 2004; Young, 2018). Within this paradigm, the reward magnitude and delay sensitivities individually predict trial-level preference: high sensitivity to when choice options would be received is associated with choosing SSs more frequently due to their immediacy, whereas high sensitivity to how much money each choice option would deliver is associated with choosing LLs more frequently due to their magnitude. If a participant’s ambivalence across trials is relatively high, however, their ability to discriminate between choice options would likely be reduced. Therefore, we further hypothesized that individuals experiencing ambivalence between the choice options would show reduced sensitivities to the options’ reward delay and magnitude differences. Specifically, as ambivalence increases, it was hypothesized (H2) that the relative impact of the reward magnitude and delay sensitivities on trial-level choices would diminish (i.e., trend toward 0).

## Materials and Methods

## Participants

Participants (*N* = 370; 79.9% White, 37.5% women, *M*_*age*_ = 35.12 years, age range: 19–65 years) who self-reported to be 18 years or older and located in the United States were recruited from the Amazon Mechanical Turk (MTurk) worker pool. To qualify, MTurk workers had to have completed at least 100 MTurk “jobs,” i.e., Human Intelligence Tasks (HITs), and to have at least a 95% HIT approval rate. Participants with these characteristics have been shown to provide higher quality data without the use of attention check questions (Peer et al., 2014).

### Measures

#### Delay Discounting Assessment

The standard 27-item MCQ (Kirby et al., 1999) presents participants with choices between SS/LL monetary rewards. SS magnitudes range from $11 to $78 and LL magnitudes range from $25 to $85; the delays for the LLs range from 7 to 186 days. Each trial is classified into a magnitude condition based on the amount of the LL, and we only used the small ($25–$35) and large ($75–$85) magnitude items, resulting in 18 trials used per participant. Each magnitude condition consists of nine trials, each of which has an associated discount rate, i.e., *k* of Mazur (1987), and can be rank ordered from 1 (lowest associated *k* value) to 9 (highest associated *k* value). See Table 1 for listing of MCQ trials used.

**TABLE 1:** Abbreviated Monetary Choice Questionnaire.

SS	LL	Delay in days	*k* at indiff.	*k*rank
34	$35	186	0.00016	1
78	$80	162	0.00016	1
28	$30	179	0.00040	2
80	$85	157	0.00040	2
22	$25	136	0.0010	3
67	$75	119	0.0010	3
25	$30	80	0.0025	4
69	$85	91	0.0025	4
19	$25	53	0.0060	5
55	$75	61	0.0060	5
24	$35	29	0.016	6
54	$80	30	0.016	6
14	$25	19	0.041	7
41	$75	20	0.041	7
15	$35	13	0.10	8
33	$80	14	0.10	8
11	$30	7	0.25	9
31	$85	7	0.25	9

Note: k at indiff. = the value of the discount rate at which the immediate and delayed rewards are of equal value; k rank = trials with the same values of k grouped in ascending rank order; SS = smaller, sooner rewards; LL = larger, later rewards. Table adapted from Kirby et al. (1999).

#### Ambivalence Measurement Conditions

Monetary Choice Questionnaire trials were adapted with four possible strategies to assess ambivalence (i.e., ambivalence measurement conditions): A1, A2, A3, and A4. Inclusion of these four conditions was exploratory, as we are aware of no previous efforts to assess trial-level ambivalence for preferences in binary choice DD tasks.

In the A1, A2, or A3 conditions, each MCQ trial was followed with a question asking the participant to indicate degree of *certainty* (A1), *unhappiness if receiving the choice they didn’t select* (A2), or *indecision* (A3; adapted from Priester and Petty, 2001). Participants in the A1, A2, and A3 conditions responded using an 11-point Likert scale ranging from 0 (*not at all certain*, *not at all unhappy, feel no indecision at all*, respectively) to 10 (*completely certain*, *completely unhappy, feel maximum indecision*, respectively). In condition A4, the binary choice trials were replaced with a 100-point continuous slider to indicate degree of relative preference between strongest preference for the SS at the far left (0th point) and strongest preference for the LL at the far right (100th point).

### Procedure

The study was administered using Qualtrics. Magnitude conditions and ambivalence measurement conditions were paired and counterbalanced such that all participants were exposed to each magnitude condition of the MCQ (small and large) *via* a different ambivalence measurement condition, termed magnitude-ambivalence pairings. Specifically, initial data collection only included the A1 and A2 conditions, whereas subsequent participants (latter half of the sample) were exposed only to the A3 and A4 conditions. This resulted in four possible magnitude-ambivalence pairings in A1/A2 (Small-A1 and Large-A2; Small-A2 and Large-A1; Large-A1 and Small-A2; Large-A2 and Small-A1) and four possible magnitude-ambivalence pairings in A3/A4 (Small-A3 and Large-A4; Large-A4 and Small-A3; Large-A3 and Small-A4; Small-A4 and Large-A3). Trials within magnitude conditions were blocked and randomized within that block. Upon completion of the study, which was estimated to take no longer than 5 min, participants were compensated the recommended pay rate requested by MTurk workers, i.e., $0.10 per minute for a total of $0.50 (Chandler and Shapiro, 2016). Participants read over an information statement before deciding to participate, and all procedures were approved by the Institutional Review Board (Human Research Protection Program) at the University of Kansas-Lawrence campus.

### Data Analysis

All data preparations and plotting were conducted using the *tidyverse* framework (Wickham et al., 2019) in the R 3.6.3 statistical environment (R Core Team, 2019). Mixed model analyses were conducted using the *lme4* package (Bates et al., 2015), and subsequent contrasts and interactions were probed using the *emmeans* package (Lenth, 2021). We report *b*, the unstandardized coefficients of our regression models.

#### Data Preparation

##### Ambivalence and Choice Scoring

Due to differences in question phrasing and scale ranges, equivalent numerical scores between the ambivalence measurement conditions did not necessarily correspond to identical degrees of ambivalence. For instance, degree of certainty (A1) refers to the exact opposite of degree of indecision (A3); moreover, a 10-point Likert scale denoting degree of certainty (A1) produces qualitatively different scores compared to a 100-point slider scale (A4) that indicates relative preference between the SS and LL. Therefore, ambivalence scores were adjusted such that the minimum possible score represents *least ambivalence*, and the maximum possible score represents *most ambivalence*. For A1 and A2, scores were flipped about the midpoint such that 0 represents least ambivalence, and 10 represents most ambivalence. For A3, the original scaling was preserved (i.e., 0 represents least ambivalence, and 10 represents most ambivalence). For A4, the raw 0–100 scale provides the relative preference of the LL to the SS (0-completely prefer SS; 100-completely prefer LL); therefore, to calculate ambivalence *via* the distance from the midpoint, each score was subtracted by 50, made an absolute value, subtracted again by 50, made again an absolute value (so that 0 represents least ambivalence, and 50 represents most ambivalence), and lastly divided by 5 to match the scale range (0–10) of the other ambivalence conditions.

##### Maximum Ambivalence and Switch Trial Computation

Within a magnitude condition for each participant, the maximum ambivalence trial was denoted as the trial with the highest ambivalence score. Trial numbers were averaged if multiple trials had the same maximum ambivalence score. To designate the trial for the switch point, we denoted the second trial around the switch in preference as the switch trial (i.e., the first trial where an LL is preferred when trials are ordered by ascending *k* rank) for participants who switched preference once across trials. For participants with multiple switch points, *k* values were computed as the discount rate most consistent with the response pattern or as the geometric mean of discount rates that were equally consistent (Gray et al., 2016). Then, the switch trial was denoted as the trial with the *k* value of the next highest *k* rank. For instance, if a response pattern yielded a 0.0019 *k* value, the switch trial would be marked as 4 according to its *k* rank (in Table 1).

#### H1: Within-Individual Ambivalence Tracks Preference Switches

Prior to any H1 analyses, trial numbers were centered within individuals such that 0 represents the switch trial. Although the location of switch trials varied between individuals and magnitude conditions, preference switches occurred most often within two trials around MCQ trial 6 by *k* rank. Thus, switch-centered trials farthest away from the 0-point had relatively few data points and high standard errors. To address this, switch-centered trials with cell counts totaling less than 20% of the participant count within each magnitude-ambivalence pairing were removed prior to H1 analyses. Moreover, if a participant never switched preference for a given magnitude condition, then that trial set was excluded from analyses because switch trials cannot be readily estimated from such response patterns (their data are shown in the Supplementary Material). For reference, 15.1 and 16.5% of our response patterns in small and large magnitude conditions, respectively, did not show a preference switch, whereas by ambivalence measurement condition, response patterns without preference switches were: A1 (16.8%), A2 (14.6%), A3 (14.1%), and A4 (17.8%).

As previously stated, it was expected that ambivalence would peak around the switch point. Initially, we attempted to fit non-linear curves (i.e., Gaussian and Cauchy) to the ambivalence scores *via* the *nlme* package in R (Pinheiro et al., 2021) due to the apparent non-linear form of the data. However, the non-linear models had convergence issues potentially due to a subset of individuals not showing the apparent non-linear form. Thus, instead of a non-linear approach, we used a dual-slopes mixed model with two linear slopes for before and after the switch trial and set the intercept on the switch trial; this intercept was chosen *via* model comparisons showing that the point of maximum ambivalence for most individuals was indeed at the switch trial (see Supplementary Material for more information on this approach).

The two slope terms were then quantified to determine (1) if ambivalence scores do indeed increase prior to the switch trial, (2) if, after a switch trial, ambivalence scores decrease again, and 3) whether there is asymmetry between slopes before versus after the switch trial. All nominal factors (magnitude condition, ambivalence measurement condition) within the model were effects coded. Random effects included both slope terms and random intercepts that were nested within individuals.

#### H2: Trial-Level Ambivalence Covaries With Diminished Sensitivities to Reward Delay and Magnitude

The goal of H2 analyses was to investigate whether greater degree of ambivalence is associated with diminished sensitivity to reward magnitude and delay differences as it relates to trial-level choice. We compared logistic mixed models following previous examples (Wileyto et al., 2004; Young, 2018) to determine if adding ambivalence variables (measurement conditions and scores) provided incremental predictive validity according to Akaike Information Criterion (AIC; Akaike, 1974). When interpreting AIC values, lower values indicate preferred models with a minimum difference of 4 required to prefer one model over another (Burnham et al., 2011). Three models were compared: (1) a “Base” model with only reward magnitude and delay sensitivity predictors (natural log-transformed LL/SS ratios) as done previously (Wileyto et al., 2004; Young, 2018), (2) a “BaseAmbMag” model with the reward sensitivities and magnitude-ambivalence pairings, and (3) an “AmbMag” model with reward sensitivities, magnitude-ambivalence pairings, and ambivalence scores. The interaction terms between the reward sensitivities and ambivalence scores would directly test H2, assuming that AmbMag is found to be the preferred model according to AIC differences. For all models, reward sensitivities were included as continuous, random effects in addition to their fixed effects. Any model terms that did not include the reward sensitivities, either as first-order terms or interactions, were removed as done previously (Young, 2018).

## Results and Discussion

### Participant Attention and Data Quality

We used the detection of the magnitude effect (Thaler, 1981; Benzion et al., 1989; Myerson and Green, 1995; Green et al., 1997; Grace and McLean, 2005), the well-established phenomenon where smaller rewards are discounted more steeply than larger rewards, as our group-level attention check. A paired samples *t*-test contrasted within-individual differences in natural log-transformed *k* values between small and large magnitude conditions, and detected a significant difference, *t*(369) = 26.35, *p* < 0.001, Cohen’s *d* = 0.32, with small magnitude rewards being discounted more steeply than large magnitude ones, *M*_*Difference*_ = 0.66, *SD* = 0.48. Although this study is limited in the lack of response validity indicators to gauge participants’ attentiveness and engagement, our replication of the well-established magnitude effect serves as our group-level attention check and provides some assurance that participants were paying attention to the survey. Moreover, only MTurk workers that had completed at least 100 HITs with at least a 95% HIT approval were eligible for this study, which has been shown to provide higher quality data (Peer et al., 2014) and has been recommended as an alternative to using attention checks (Chandler and Shapiro, 2016). Additionally, when averaging consistency estimates between magnitude conditions, 79.2% of our participants had perfect consistency (one switch point per magnitude condition). Some researchers have suggested consistency scores serve as a proxy for attentiveness (Gray et al., 2016), so we believe the majority of our participants paid attention to and understood the task. Finally, there have been reports of “poorer data quality” in MTurk studies because of the presence of non-United States participants who may be hiding their IP address and subsequent geolocation (for a discussion, refer to Kennedy et al., 2020). Our data suggest that 2.9% of our sample consisted of participants with IP addresses outside the United States, whereas 7.3% of our sample used a virtual private network (VPN) to mask their geolocation. We elected not to remove these participants because (1) we believed the proportion of non-US participants was sufficiently small and unlikely to impact our results and (2) it is not uncommon for many United States participants to use a VPN service (Security.org Team, 2021). Moreover, even amongst the literature suggesting that a higher proportion of data from individuals using VPN is “poorer quality” (e.g., Kennedy et al., 2020), the absolute rate of “poorer quality” data remains low.

### H1: Within-Individual Ambivalence Tracks Preference Switches

**FIGURE 1:**
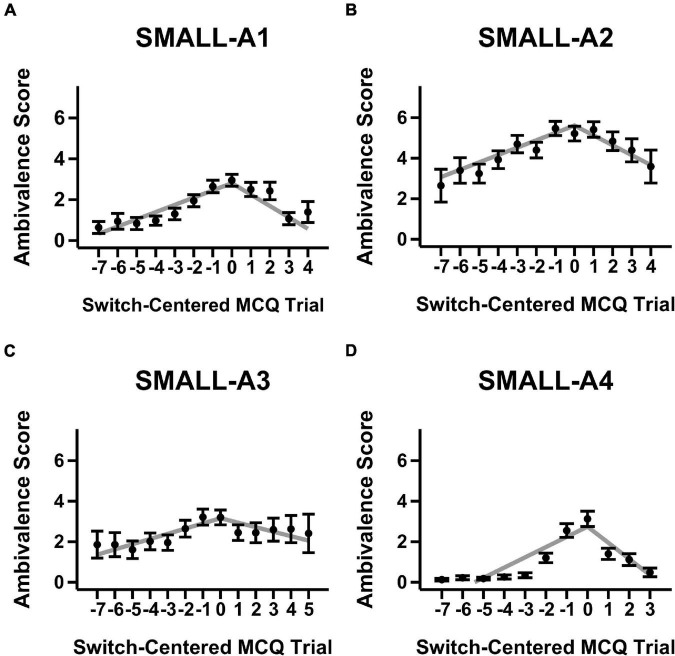
Mean ambivalence scores and dual-slopes mixed model predictions for small magnitude trials split by ambivalence measurement condition. X-axis denotes the MCQ trial number centered by switch trial, Y-axis denotes the degree of ambivalence, and the panels denote the specific magnitude-ambivalence pairing: **(A)** Small-A1; **(B)** Small-A2; **(C)** Small-A3; **(D)** Small-A4. Black points indicate the trial-level means in self-reported ambivalence scores with standard error bars. Gray lines indicate the predicted ambivalence scores from the dual-slopes mixed model.

**FIGURE 2:**
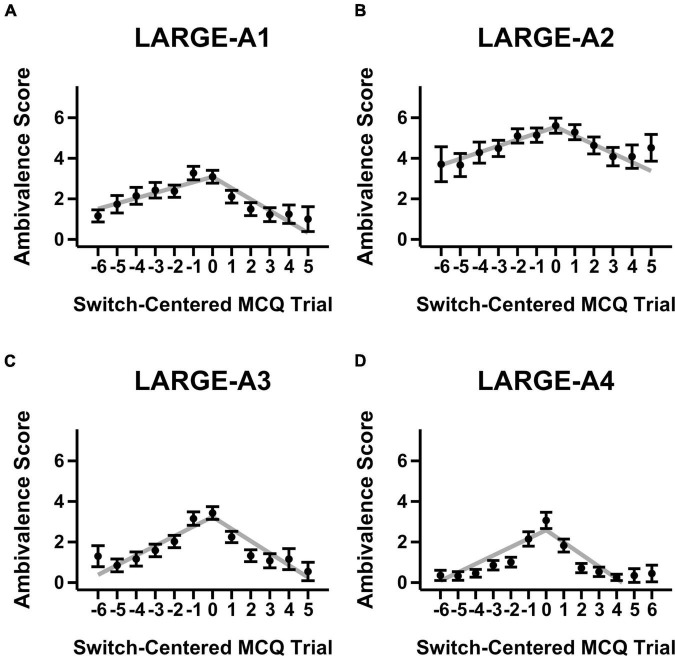
Mean ambivalence scores and dual-slopes mixed model predictions for large magnitude trials split by ambivalence measurement condition. X-axis denotes the MCQ trial number centered by switch trial, Y-axis denotes the degree of ambivalence, and the panels denote the specific magnitude-ambivalence pairing: **(A)** Large-A1; **(B)** Large-A2; **(C)** Large-A3; **(D)** Large-A4. Black points indicate the trial-level means in self-reported ambivalence scores with standard error bars. Gray lines indicate the predicted ambivalence scores from the dual-slopes mixed model.

Figures 1, 2 show mean ambivalence scores centered on switch trials across magnitude-ambivalence pairings overlaid with dual-slopes mixed model predictions. Visual inspection of the figures suggests qualitative support for H1. That is, when centered on participants’ respective switch trials, ambivalence peaks around and steadily declines away from the switch trial. However, while the aggregate data took on an apparent non-linear form, many participants also showed constant ambivalence across trials (8.7–29.9% of individuals depending on the magnitude-ambivalence pairing; see Supplementary Figure 3 in the Supplementary Material for exemplar ambivalence score patterns). This between-subject variability may have led to the non-linear models described in section “Materials and Methods” failing to converge. As such, a dual-slopes linear mixed model quantified the apparent trends utilizing slope terms for ambivalence scores both before and after the switch trial.

The results from the dual-slopes linear mixed model overall supported the H1 hypothesis (see Table 2). Across magnitude-ambivalence pairings, ambivalence scores increased prior to the switch trial (*b* = 0.37, 95% CI = [0.32, 0.42], SEM = 0.02, *p* < 0.001) and decreased after the switch trial (*b* = −0.53, 95% CI = [−0.60, −0.46], *SEM* = 0.04, *p* < 0.001). The decreases in ambivalence scores following the switch trial were also sharper than the increases preceding it (*p*s < 0.001); however, post-switch slopes were likely steeper due to the right side of the switch trial on the X-axis containing more trials across pairings (see Figures 1, 2). All slope values between pairings were significantly different from 0 (positive before switch, negative after switch; *p*s < 0.001), meaning each ambivalence measurement condition seemed to adequately characterize degree of ambivalence across the switch-centered MCQ trials. Given the multiple ambivalence measurement conditions, a secondary question of interest was concerned with identifying the condition that characterized ambivalence with highest sensitivity. However, the data showed minimal differences between conditions: the only significant comparison was A4 having a more negative slope after switch compared to A3 (*p* < 0.01). In that regard, ambivalence measurement conditions showed relatively consistent sensitivity in characterizing ambivalence scores for switch-centered trials. Additionally, ambivalence score means for switch-centered trials (Figures 1, 2) were relatively low and close to score means for trial sets where participants did not switch preference (Supplementary Figures 1, 2). Although this observation is important to note, we believe the lack of within-individual variation in participants who did not switch preference provides further support for H1 and that the relevant comparison is the constant versus variable ambivalence for trial sets without a switch trial and those with one, respectively.

**TABLE 2:** Parameter estimates of the dual-slopes linear mixed model of ambivalence scores.

	Fixed effects				
Parameter	*b*	95% CI	SE	*z*/*t*	*p*
**(Intercept)**	**3.60**	**[3.36, 3.84]**	**0.12**	**29.57**	**<0.001**
SmallMag	–0.02	[–0.12, 0.09]	0.05	–0.30	0.76
**A1**	**−−0.65**	**[−0.93, −0.37]**	**0.14**	**−−4.52**	**<0.001**
**A2**	**1.98**	**[1.70, 2.26]**	**0.14**	**13.78**	**<0.001**
**A3**	**−−0.40**	**[−0.68, −0.12]**	**0.14**	**−−2.78**	**0.01**
**Pre-Switch**	**0.37**	**[0.32, 0.42]**	**0.02**	**14.77**	**<0.001**
**Post-Switch**	**−−0.53**	**[−0.60, −0.46]**	**0.04**	**−−14.76**	**<0.001**
SmallMag × A1	–0.12	[−0.47, 0.23]	0.18	–0.67	0.50
SmallMag × A2	0.05	[−0.30, 0.40]	0.18	0.28	0.78
SmallMag × A3	–0.02	[−0.38, 0.33]	0.18	–0.14	0.89
SmallMag × Pre-Switch	–0.002	[−0.03, 0.04]	0.02	0.07	0.95
SmallMag × Post-Switch	0.01	[−0.04, 0.07]	0.03	0.51	0.61
A1 × Pre-Switch	–0.06	[−0.13, 0.01]	0.04	–1.68	0.09
A1 × Post-Switch	–0.02	[−0.13, 0.08]	0.05	–0.46	0.65
A2 × Pre-Switch	–0.03	[−0.10, 0.04]	0.04	–0.83	0.40
A2 × Post-Switch	0.07	[−0.04, 0.18]	0.05	1.28	0.20
A3 × Pre-Switch	–0.001	[−0.07, 0.07]	0.04	–0.06	0.95
**A3 × Post-Switch**	**0.12**	**[0.02, 0.22]**	**0.05**	**2.38**	**0.02**
SmallMag × A1 × Pre-Switch	0.04	[−0.03, 0.12]	0.04	1.13	0.26
SmallMag × A2 × Pre-Switch	0.02	[−0.05, 0.10]	0.04	0.59	0.55
**SmallMag × A3 × Pre-Switch**	**−−0.11**	**[−0.19, −0.03]**	**0.04**	**−−2.82**	**0.05**
SmallMag × A1 × Post-Switch	–0.02	[−0.13, 0.10]	0.06	–0.31	0.76
SmallMag × A2 × Post-Switch	–0.04	[−0.16, 0.07]	0.06	–0.71	0.48
**SmallMag × A3 × Post-Switch**	**0.17**	**[0.06, 0.28]**	**0.06**	**3.11**	**0.002**

*SmallMag, small magnitude condition; Pre-Switch, slope term before switch point (i.e., before switch); Post-Switch, slope term after switch point (i.e., after switch); A1, Ambivalence measurement condition 1; A2, Ambivalence measurement condition 2; A3, Ambivalence measurement condition 3; 95% CI reflect Wald confidence intervals. Significant effects in bold.*

Overall, H1 was supported in that ambivalence scores tended to vary across trials and track switches in preferences. Our study shows that participant-reported ambivalence scores peak at the switch trial and steadily decrease away from it with minimal differences between magnitude-ambivalence pairings, which to the authors’ knowledge is the first study to validate this within an assessment of DD. These findings parallel those of studies using mouse cursor trajectories (Dshemuchadse et al., 2013) and response times (Robles and Vargas, 2007, 2008; Rodriguez et al., 2014) to explore decision making around the point of subjective value equivalence, which show correlates of greater choice deliberation. While these convergent findings are interesting, it is presently unclear whether cursor trajectories or RTs merely covary with ambivalence scores or directly map onto the ambivalence construct. Regardless, the demonstrated variability in ambivalence scores and relation to trials associated with the discount rate allowed us to investigate how ambivalence factors in trial-level decision making in H2.

### H2: Trial-Level Ambivalence Covaries With Diminished Sensitivities to Reward Delay and Magnitude

H1 revealed that within-individual ambivalence tracked switches in preference across DD trials. H2 sought to extend H1 by testing associations between trial-level ambivalence and sensitivities to reward magnitudes and delays. We first compared predictive utility based on AIC scores of an omnibus model including reward sensitivities, magnitude-ambivalence pairings, and ambivalence scores (AmbMag) to a model with only the reward sensitivities and magnitude-ambivalence pairings (BaseAmbMag) as well as a model with only the reward sensitivities (Base). Overall, the AmbMag model (omnibus; AIC = 4112.0) had substantially improved predictive utility compared to the BaseAmbMag model (AIC = 4159.0, ΔAIC = 47 versus AmbMag), which itself evinced substantially improved predictive utility compared to the Base model (AIC = 4306.1, ΔAIC = 147.1 versus BaseAmbMag). The results therefore warrant that adding ambivalence estimates to models predicting DD choices improves model accuracies.

The omnibus AmbMag model estimates are shown in Table 3. The effects of reward magnitude (*OR* = 420836.64, *b* = 12.95, 95% CI = [11.01, 14.90], SEM = 0.99, *p* < 0.001) and delay (*OR* = 0.29, *b* = −1.25, 95% CI = [−1.38, −1.12], SEM = 0.07, *p* < 0.001) both significantly modulated DD choices as shown previously (Young, 2018). Specifically, as the magnitude differences between choice options increasingly favored the LL option, so too did trial choices. Conversely, as the LL became increasingly delayed relative to the SS, choice allocations favored the SS. Moreover, the reward delay sensitivity was found to depend on the magnitude condition (*OR* = 0.90, *b* = −0.11, 95% CI = [−0.16, −0.06], SEM = 0.03, *p* < 0.001), such that participants were more sensitive to the reward delay differences in the small magnitude condition compared to the large magnitude one (*p* < 0.001). This interaction reflects what is commonly referred to as the “magnitude effect” within DD research (Thaler, 1981; Benzion et al., 1989; Myerson and Green, 1995; Green et al., 1997; Grace and McLean, 2005), and serves as further evidence that reward magnitude is a key dimension in DD decision making.

**TABLE 3:** Parameter estimates of the logistic mixed model of ambivalence and sensitivities to delay and magnitude ratio.

	Fixed Effects					
Parameter	*OR*	*b*	95% CI	SE	*z*/*t*	*p*
AmbMag Model						
**MagRatio**	**420836.64**	**12.95**	**[11.01,14.90]**	**0.99**	**13.05**	**<0.001**
**DelayRatio**	**0.29**	**−1.25**	**[−1.38, −1.12]**	**0.07**	**−18.42**	**<0.001**
MagRatio × SmallMag	0.65	−0.43	[−0.89,0.03]	0.23	−1.84	0.065
**DelayRatio × SmallMag**	**0.90**	**−0.11**	**[−0.16, −0.06]**	**0.03**	**−4.01**	**<0.001**
MagRatio × A1	0.84	−0.18	[−2.67,2.31]	1.27	−0.14	0.89
MagRatio × A2	0.31	−1.16	[−3.73,1.40]	1.31	−0.89	0.37
MagRatio × A3	1.40	0.34	[−0.92,1.61]	0.65	0.53	0.59
DelayRatio × A1	0.94	−0.06	[−0.19,0.07]	0.07	−0.92	0.36
DelayRatio × A2	1.02	0.02	[−0.12,0.15]	0.07	0.22	0.82
DelayRatio × A3	1.05	0.05	[−0.08,0.18]	0.07	0.78	0.44
MagRatio × AmbScore	0.90	−0.10	[−0.24,0.03]	0.07	−1.49	0.14
**DelayRatio × AmbScore**	**1.03**	**0.03**	**[0.01,0.04]**	**0.01**	**3.81**	**<0.001**
MagRatio × SmallMag x A1	1.16	0.15	[−1.60,1.90]	0.89	0.17	0.87
MagRatio × SmallMag x A2	0.55	−0.60	[−2.41,1.20]	0.92	−0.65	0.51
MagRatio × SmallMag x A3	1.08	0.08	[−1.63,1.79]	0.87	0.10	0.92
DelayRatio × SmallMag x A1	1.00	0	[−0.16,0.17]	0.08	0.05	0.95
DelayRatio × SmallMag x A2	1.13	0.12	[−0.05,0.30]	0.09	1.41	0.16
DelayRatio × SmallMag x A3	1.16	0.15	[−0.01,0.31]	0.08	1.81	0.07
MagRatio × SmallMag × AmbScore	0.98	−0.02	[−0.13,0.09]	0.06	−0.36	0.72
DelayRatio × SmallMag × AmbScore	1.01	0.01	[−0.01,0.02]	0.01	1.09	0.27
**MagRatio × AmbScore × A1**	**0.78**	**−0.25**	**[−0.47, −0.03]**	**0.11**	**−2.20**	**0.03**
MagRatio × AmbScore × A2	1.12	0.11	[−0.08,0.31]	0.10	1.15	0.25
MagRatio × AmbScore × A3	1.03	0.03	[−0.16,0.23]	0.10	0.33	0.74
DelayRatio × AmbScore × A1	1.00	0	[−0.02,0.02]	0.01	−0.04	0.97
DelayRatio × AmbScore × A2	0.99	−0.01	[−0.03,0.01]	0.01	−1.33	0.18
**DelayRatio × AmbScore × A3**	**0.97**	**−0.03**	**[−0.05, −0.01]**	**0.01**	**−3.02**	**0.002**
MagRatio × SmallMag × A1 × AmbScore	0.91	−0.09	[−0.34,0.15]	0.13	−0.75	0.45
MagRatio × SmallMag × A2 × AmbScore	1.06	0.06	[−0.16,0.28]	0.11	0.53	0.59
MagRatio × SmallMag × A3 × AmbScore	1.21	0.19	[−0.004,0.39]	0.01	1.92	0.054
DelayRatio × SmallMag × A1 × AmbScore	1.02	0.02	[−0.001,0.05]	0.01	1.98	0.057
**DelayRatio × SmallMag × A2 × AmbScore**	**0.96**	**−0.04**	**[−0.06, −0.01]**	**0.01**	**−3.14**	**0.002**
DelayRatio × SmallMag × A3 × AmbScore	0.99	−0.01	[−0.03,0.01]	0.01	−1.08	0.28

*MagRatio, sensitivity to magnitude differences; DelayRatio, sensitivity to delay differences; A1, Ambivalence condition 1; A2, Ambivalence condition 2; A3, Ambivalence condition 3; OR, odds ratio. 95% CI reflect Wald confidence intervals. Significant effects in bold.*

Across magnitude-ambivalence pairings, the effect of reward delay sensitivity (*OR* = 1.03, *b* = 0.03, 95% CI = [0.01, 0.04], SEM = 0.01, *p* < 0.001) on trial-level choice decreased as ambivalence scores increased (i.e., increasing ambivalence trended delay sensitivity values toward 0). In other words, the delay to the LL seemed to weigh less in participants’ decision making when they were less certain about their preference. While we observed significant interactions between reward delay sensitivity, ambivalence scores, magnitude condition, and ambivalence measurement condition (see Table 3), we choose not to interpret these effects as (1) H1 analyses showed all magnitude-ambivalence pairings to consistently characterize trends in ambivalence scores and (2) we had no *a priori* hypotheses regarding differences between the ambivalence measurement conditions.

That ambivalence scores covary with reduced sensitivities to delays between choice options demonstrates a novel finding in DD research. Nonetheless, H2 is partially supported in that participants’ sensitivity to reward magnitude differences does not seem to vary even as their choice ambivalence increases, and they may also look to features other than the delays between choice options when making their decision. However, what features may become more prominent during states of ambivalence is left to future research.

### Limitations and Future Directions

It is necessary to acknowledge that the primary limitation of our study is the use of hypothetical outcomes for our DD assessment. However, prior research has shown statistically equivalent effects when using real versus hypothetical rewards for these assessments (Matusiewicz et al., 2013). A broader limitation of our study is the use of the MCQ as our chosen DD assessment. Although it is a popular task for assessing DD, some have criticized its fixed-choice structure as lacking in adequate sampling of the possible parameter space of reward magnitudes and delays (Young, 2018). For instance, while the range of magnitude differences is $1–$54 (translates to 0–1 in natural log transformed magnitude ratio between LL/SS), the range of delays to LL receipt is 7–186 days (translates to 2–6 in natural log transformed delay ratio between LL/SS). Hence, it is unclear how ambivalence may track participants’ choice patterns given an alternative DD assessment. Future research may consider alternative assessments and models of DD to study choice ambivalence, including ones that incorporate each trial-level decision to model discounting behavior (Dai and Busemeyer, 2014; Rodriguez et al., 2014; Dai et al., 2016; Molloy et al., 2020; Kvam et al., 2021).

Furthermore, the present study is limited in the lack of response validity indicators to gauge attentiveness at the level of individual participants (for a discussion on response validity indicators, see Chmielewski and Kucker, 2020). While we believe participants demonstrated sufficient attentiveness on a group level, we cannot rule out the possibility that the potential inclusion of participants that might have otherwise failed response validity indicators impacted our model estimates. Participants who used a VPN or had a non-United States IP address may have been more likely to fail such indicators and bias our estimates further. This project also did not include a comparator condition to assess whether inquiries about ambivalence impacted rate of DD, nor did it include potentially less reactive measures of ambivalence, such as trial-level RT as in previous work (Robles and Vargas, 2007, 2008; Rodriguez et al., 2014).

A direction for future research would be to directly assess the convergence between non-reactive (e.g., response time) and reactive (e.g., mouse cursor trajectory, self-reported ambivalence scores) measures relevant to choice difficulty. One idea that we propose might be particularly worthwhile is a construct we call the *window of ambivalence*. Ambivalence is relatively high for several trials around the switch trial when observing Figures 1, 2. This range of relatively high ambivalence scores may be termed the “window of ambivalence,” which has not been identified in previous research. We attempted to index this window by assessing the spread parameters from non-linear distribution curve fits to ambivalence data across trials. However, similar to our previous non-linear modeling attempts, the models had convergence issues that deemed the analysis plan untenable (see Supplementary Material for more information on this approach). Future research may wish to expand upon this work through assessing the true functional form of the window of ambivalence. Then, researchers could include manipulations of DD (e.g., Radu et al., 2011) to see whether the ambivalence window tracks the change in discounting rate along the trial continuum (such as in the MCQ) and whether the window expands or shrinks following the manipulation.

In conclusion, we used largely novel assessment strategies to characterize trial-level ambivalence in a DD task. On a group-level, our results showed that: (1) ambivalence tracks preference switches across trials; and (2) ambivalence is associated with a reduced ability to discriminate between reward delays when it comes to trial-level choice. We believe that ambivalence may be an interesting construct to explore further in research on DD choice and manipulations of individual rates of DD.

## Data Availability Statement

The raw data supporting the conclusions of this article will be made available by the authors, without undue reservation.

## Ethics Statement

The studies involving human participants have been reviewed and approved by the Institutional Review Board (Human Research Protection Program) at the University of Kansas-Lawrence campus.

## Author Contributions

RY and SG contributed to the conception and designed of the study. SG organized the database and wrote the first draft of the manuscript. SG and AS performed the statistical analyses. All authors contributed to manuscript revision, read, and approved the submitted version.

## Conflict of Interest

The authors declare that the research was conducted in the absence of any commercial or financial relationships that could be construed as a potential conflict of interest.

## Publisher’s Note

All claims expressed in this article are solely those of the authors and do not necessarily represent those of their affiliated organizations, or those of the publisher, the editors and the reviewers. Any product that may be evaluated in this article, or claim that may be made by its manufacturer, is not guaranteed or endorsed by the publisher.
