# Supplementary material for: Are You Sure: Preference and Ambivalence in Delay Discounting
Source: Front Behav Neurosci. 2022 Jan 24;15:782991. doi: 10.3389/fnbeh.2021.782991 (PMC8818853; doi:10.3389/fnbeh.2021.782991)
Supplement: Supplementary file 1 [file Data_Sheet_1.PDF]

Supplementary Material for Are You Sure: Preference and Ambivalence in Delay Discounting

1 Ambivalence Trends for Trial Sets Without Preference Switches

We ran a linear mixed model to determine the ambivalence score patterns across non-switch centered MCQ trials by *k* rank for trials sets without a switch in preference. All nominal factors (magnitude condition, ambivalence measurement condition) within the model were effects coded. Random effects included the trial number and random intercepts that were nested within individuals.

Figures S1 and S2 show mean ambivalence scores overlaid with linear mixed model predictions for ambivalence-magnitude pairings where participants reported consistent preference (either for SSs or LLs) across all trials within the respective pairing. Mean scores seem to suggest constant ambivalence across trials compared to Figures 1 and 2 in the main text. This observation was confirmed by the linear mixed model, such that the base effect of MCQ trial number on ambivalence scores was credibly flat (p = .65) and did not differ between ambivalence-magnitude pairings (ps > .30), thereby confirming constant ambivalence across trials. This finding is in line with H1 that ambivalence tracks switches in preference (i.e., ambivalence peaks around switch trial), which for "non-switchers" does not hold given the lack of an identifiable switch trial. However, a direct comparison to "switchers" is inappropriate given that ambivalence scores were switch-centered for the latter figures, meaning that within-individual variability is more directly observable given the centering.

2 Example Plot of Subject-Level Ambivalence Scores Across MCQ Trials

Figure S3 shows data for three trial sets that represent larger trends in the data. That is, a group of participants showed a response pattern consistent with the mean ambivalence scores in Figures 1 and 2 (akin to pattern of black circles), another group reported constant ambivalence across trials (akin to pattern of dark-gray triangles), and yet another group had relatively chaotic responding across the switch-centered MCQ trials (akin to pattern of light-gray squares).

3 Point of Maximum Ambivalence Analysis

To quantify the trends in ambivalence scores across trials, we first tested for the location of the trial of maximum ambivalence. To do this, we used a nonlinear Gaussian function on the frequency distributions of switch-centered maximum ambivalence trials with means occurring either at the switch trial (0-mean fit), one trial before it ([-1]-mean fit), or one trial after it ([+1]-mean fit). Table S1 provides the AIC values of nonlinear Gaussian fits for each magnitude-ambivalence pairing. Across all pairings, the 0-mean fit was much more likely than the [+1]-mean fit, with the smallest AIC difference being ≈ 47 in the Small-A3 comparison, which suggests essentially no support for any of the [+1]-mean fits (Burnham et al., 2011). In contrast, AIC differences between the 0- and [-1]-mean fits suggest the 0-mean fit to be the preferred one at best (e.g., ΔAIC ≈ 6 for the Large-A3 comparison) and essentially no difference at worst (e.g., ΔAIC ≈ 0 for the Small-A4 comparison).

The 0-mean fit, i.e., the one with maximum ambivalence occurring at switch trials, generally has

stronger support than fits with ambivalence peaking one trial to the left or right of the switch trial, respectively. However, a significant number of the model comparisons yielded the (-1)-mean fit just as likely as the 0-mean fit. One potential explanation is that maximum ambivalence trials tended to occur slightly more frequently to the left of the switch point (see Figures S4 and S5), thereby biasing the mean of the data-derived density plots. However, given that the true switch point likely occurs between the respective SS- and LL-preferred trials surrounding it, this result may be unsurprising. That is, a participant may feel maximum ambivalence right before or after switching preference, and it would have been equally defensible to have denoted the SS trial as the switch trial instead of the LL trial as was done here.

4 Quantifying the Window of Ambivalence

As stated in the *Method* section, we attempted to fit nonlinear curves to the ambivalence scores to find the best-fitting functional form of the data. Our goal was to compare Gaussian and Cauchy distribution fits, which would more intuitively test the hypothesis that ambivalence peaks around the switch trial. Namely, both the Gaussian and Cauchy distributions have the mean and location parameter, respectively, at which point the function peaks out. Using nonlinear mixed modeling would then test whether the mean (or ‘location’) parameters are significantly different from 0 (thereby directly testing if ambivalence peaks around the switch trial). Moreover, this analysis would also yield a standard deviation (in the case of Gaussian) or spread (in the case of Cauchy) parameter, which would serve to estimate the width of the “window of ambivalence.” To more conceptually verify whether the window of ambivalence has theoretical merit, our plan was to correlate the subject-level standard deviation/spread parameters with the subject-level reward magnitude and delay sensitivities from the H2 analyses, given that individuals with relatively high ambivalence only around the switch point and steeply decreasing away from it (i.e., tight window of ambivalence) would likely be able to better discriminate between rewards across relevant dimensions compared to individuals with relatively constant ambivalence across trials (i.e., wide window of ambivalence).

5 Figures

5.1 Figure S1

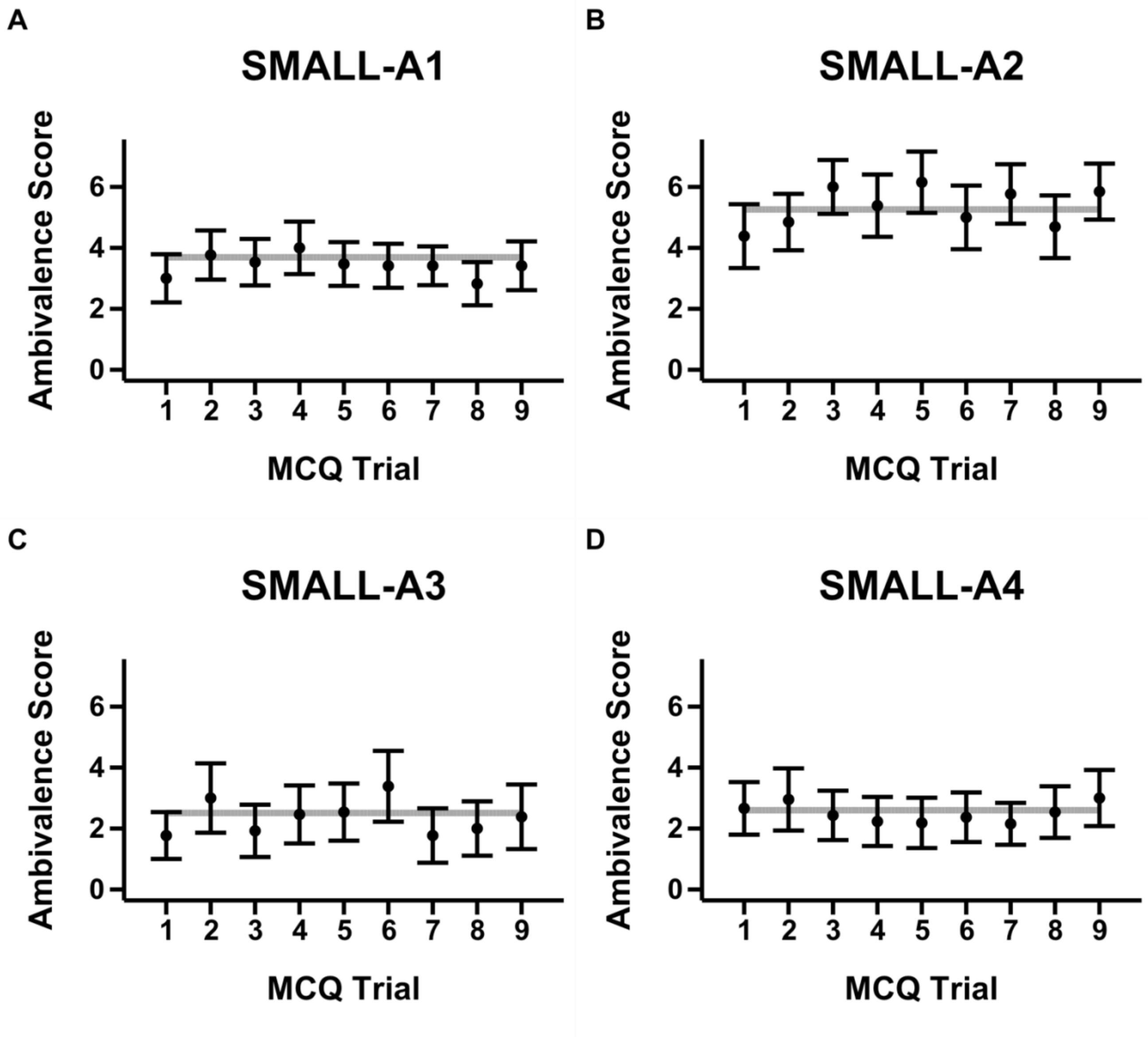

Figure S1. Mean ambivalence scores and linear mixed model predictions for **small magnitude** trials split by ambivalence measurement condition. X-axis denotes the MCQ trial number by *k* rank, Y-axis denotes the degree of ambivalence, and the panels denote the specific magnitude-ambivalence pairing. Black points indicate the trial-level means in self-reported ambivalence scores with standard error bars. Gray lines indicate the predicted ambivalence scores from the linear mixed model.

5.2 Figure S2

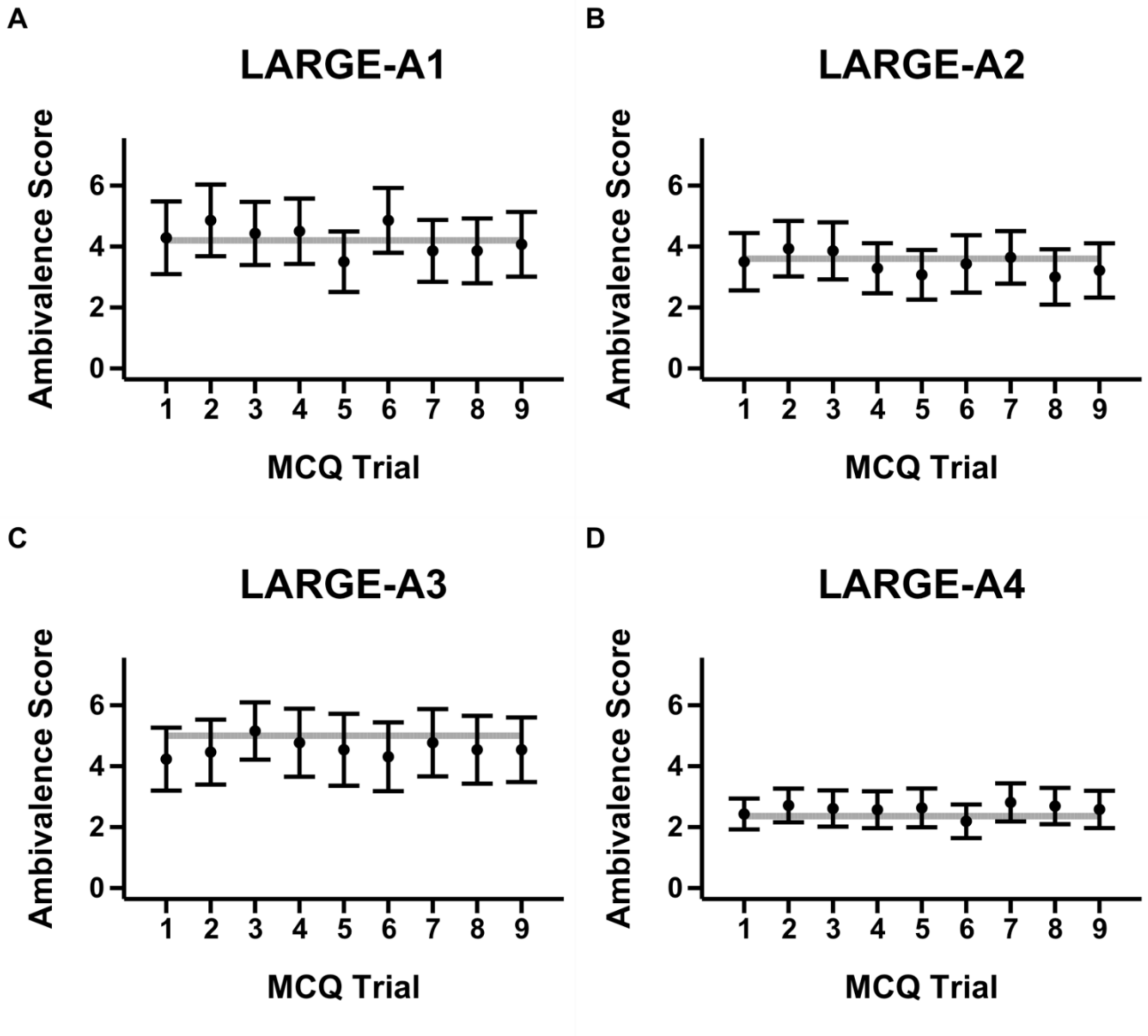

Figure S2. Mean ambivalence scores and linear mixed model predictions for **large magnitude** trials split by ambivalence measurement condition. X-axis denotes the MCQ trial number by *k* rank, Y-axis denotes the degree of ambivalence, and the panels denote the specific magnitude-ambivalence pairing. Black points indicate the trial-level means in self-reported ambivalence scores with standard error bars. Gray lines indicate the predicted ambivalence scores from the linear mixed model.

5.3 Figure S3

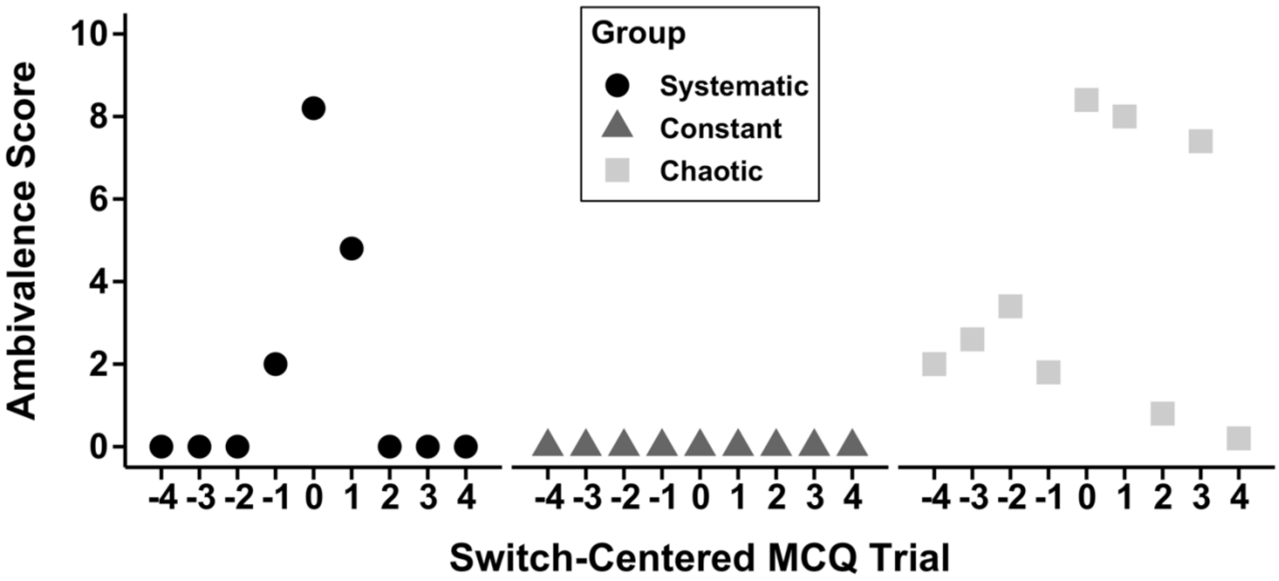

Figure S3. Exemplar ambivalence score patterns. X-axis denotes the MCQ trial number centered by switch trials, Y-axis denotes the degree of ambivalence, and the point shape denotes the ambivalence score pattern: black circles denote a pattern consistent with those in Figures 1 and 2; dark-gray triangles denote a constant ambivalence pattern; and light-gray squares denote a relatively chaotic response pattern across trials.

5.4 Figure S4

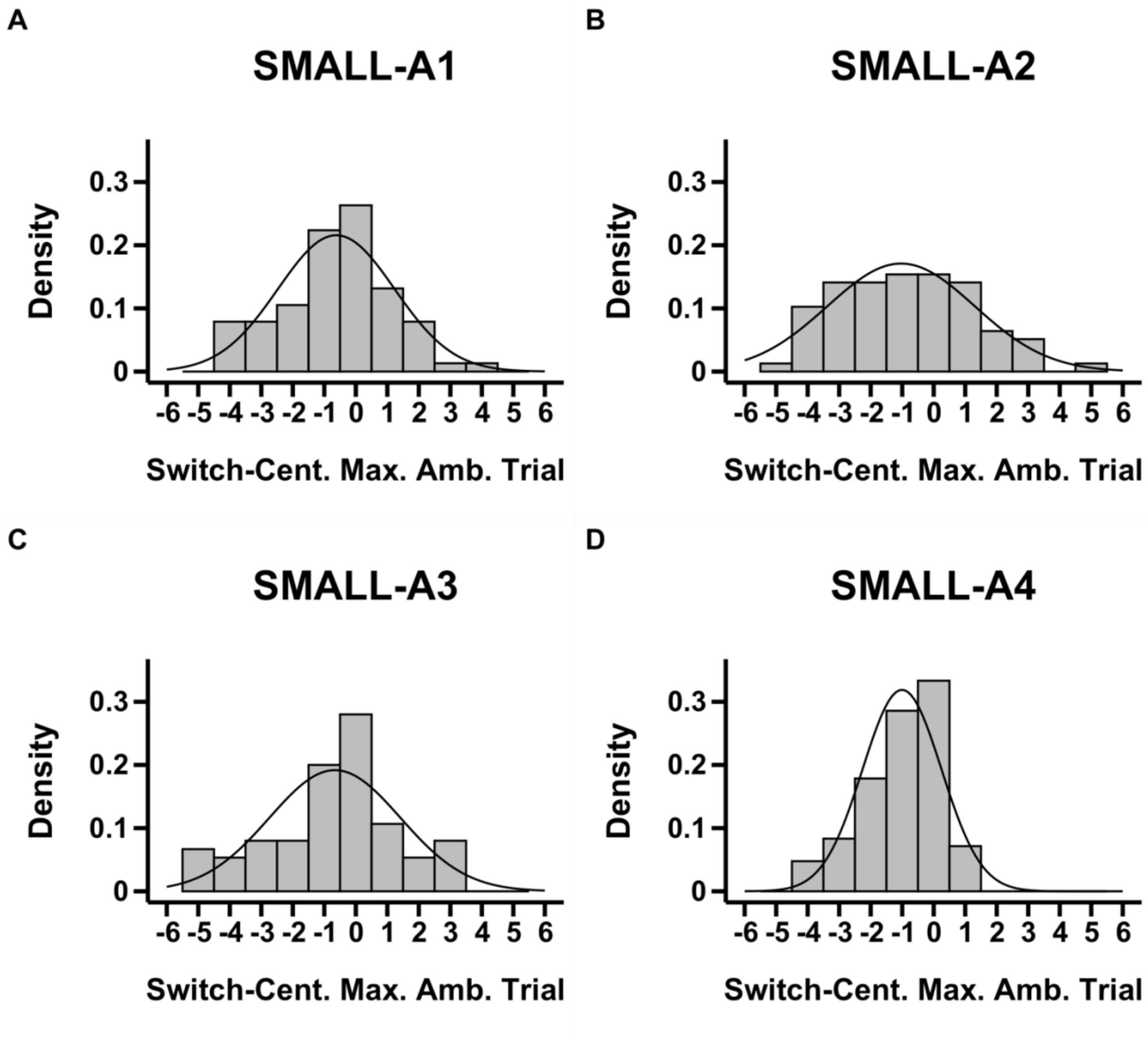

Figure S4. Density plots of switch-centered maximum ambivalence trials for **small magnitude** trials. The X-axis denotes the MCQ trial number of the maximum ambivalence trial centered by switch trials, the Y-axis denotes the density, and the panels denote the specific magnitude-ambivalence pairing. Density distributions, which simply take the frequency of each cell and divide it by the total number of observations, were derived using sample mean and standard error to allow for normal curve plotting in addition to the data.

5.5 Figure S5

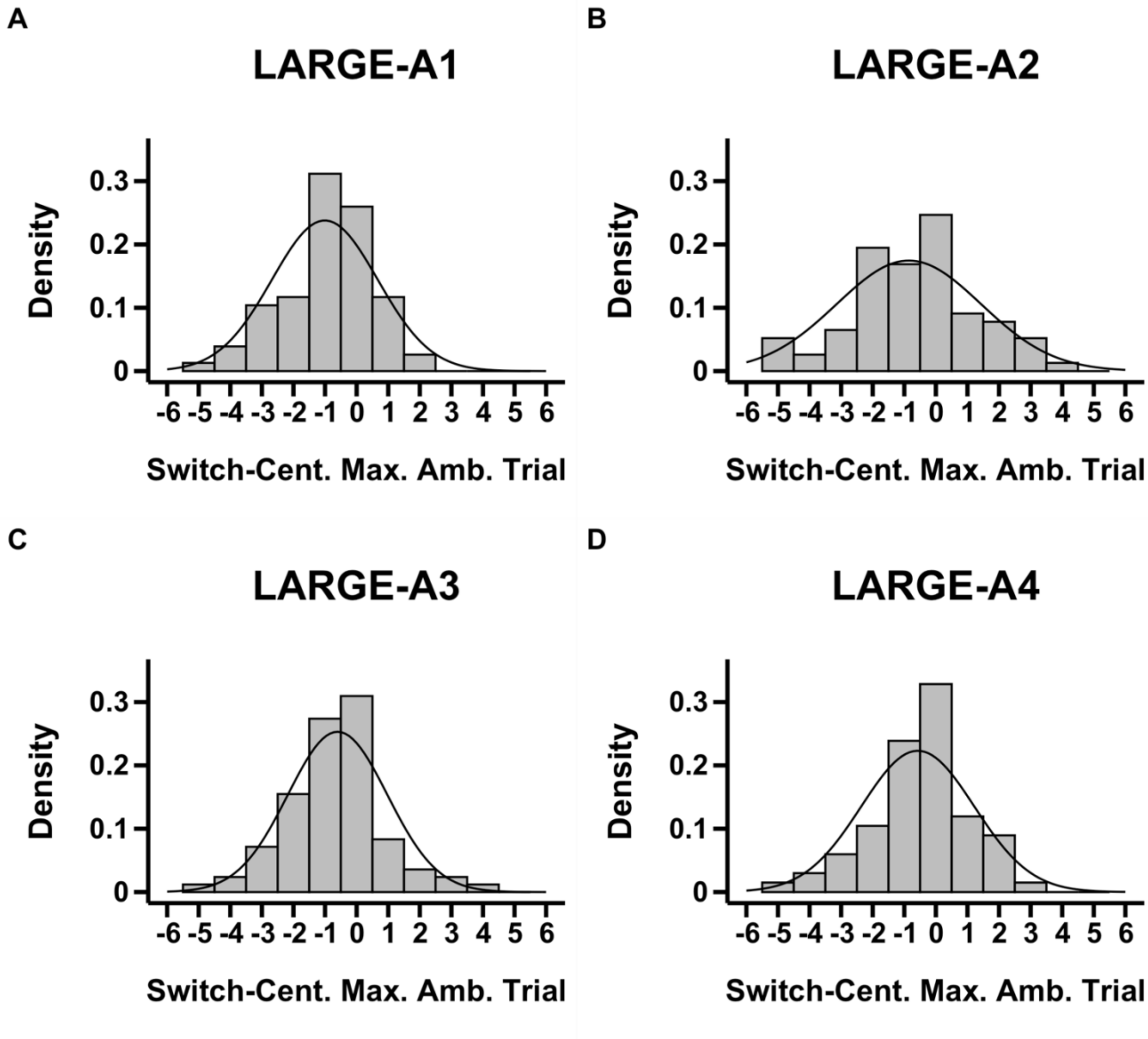

Figure S5. Density of switch-centered maximum ambivalence trials for **large magnitude** trials. The X-axis denotes the MCQ trial number of the maximum ambivalence trial centered by switch trials, the Y-axis denotes the density, and the panels denote the specific magnitude-ambivalence pairing. Density distributions, which simply take the frequency of each cell and divide it by the total number of observations, were derived using sample mean and standard error to allow for normal curve plotting in addition to the data.

6 Tables

6.1 Table S1

Table S1. AIC Values for Best-Fitting Models of Switch-Centered Maximum Ambivalence Trials

	Model		
Pairing	0-mean	(+1)-mean	(-1)-mean
SmallA1	**311.75**	362.55	316.08
SmallA2	356.48*	407.56	356.73
SmallA3	328.34*	375.09	330.35
SmallA4	279.61*	479.23	279.63
LargeA1	304.10*	404.76	304.19
LargeA2	351.42*	401.92	352.04
LargeA3	**314.13**	396.90	320.43
LargeA4	**266.51**	316.64	272.53

Note. AIC = Akaike Information Criterion 0-mean model against (+1)-mean and (-1) mean models (lower values indicate better evidence for the 0-mean model). Small-A1 = small magnitude-ambivalence condition 1 pairing; Small-A2 = small magnitude-ambivalence condition 2 pairing; Small-A3 = small magnitude-ambivalence condition 3 pairing; Small-A4 = small magnitude-ambivalence condition 4 pairing; Large-A1 = large magnitude-ambivalence condition 1 pairing; Large-A2 = large magnitude-ambivalence condition 2 pairing; Large-A3 = large magnitude-ambivalence condition 3 pairing; Large-A4 = large magnitude-ambivalence condition 4 pairing. Models in **bold** denote the model with an AIC difference of at least 4 compared to the other models and models with an asterisk* denote the model with the technically smallest AIC score that is not at least greater than 4 when compared to the AIC scores of other models within each row.
